# Applying Social Practice Theory to Explore Australian Preschool Children’s Oral Health

**DOI:** 10.1177/23800844241235615

**Published:** 2024-04-16

**Authors:** A. Durey, P. Ward, E. Haynes, S.R. Baker, H. Calache, L. Slack-Smith

**Affiliations:** 1 School of Population and Global Health, The University of Western Australia, Perth, Western Australia, Australia; 2 Research Centre for Public Health, Equity and Human Flourishing, Torrens University, Adelaide, SA, Australia; 3 School of Clinical Dentistry, Sheffield University, Sheffield, UK; 4 La Trobe University, Department of Clinical Sciences, La Trobe Rural Health School, Bendigo, VIC, Australia

**Keywords:** qualitative research, prevention and control, dental caries, pathology, social determinants of health, social theory

## Abstract

**Introduction:**

Despite substantial research and provision of dental care, significant morbidity remains for children’s oral health. Guided by social practice theory (SPT), this research moves away from the often-ineffective focus on changing individual behavior to rethinking the centrality of the social world in promoting or undermining oral health outcomes. We define social practice as a routinized relational activity linking and integrating certain elements (competence, materials, and meanings) into the performance of a practice that is reproduced across time and space.

**Objective:**

To investigate oral health in preschool children in Perth, Western Australia, using social practice theory.

**Methods:**

With no definitive methodology for investigating SPT, we chose focused ethnography as a problem-focused, context-specific approach using mainly interviews to investigate participants’ experience caring for their children’s oral health. The focus of analysis was the practice of oral health care, not individual behavior, where themes identified from participants’ transcripts were organized into categories of elements and performance.

**Results:**

Eleven parents, all of whom were married or partnered, were interviewed in 2021. Findings identified social practices relevant to oral health within parenting and family relations linked to routine daily activities, including shopping, consumption of food and beverages, and toothbrushing. Oral health literacy was reflected in integrating competence, materials, and meanings into performing oral health care, notably preferences for children to drink water over sugary beverages and information often being sourced from social media and mothers’ groups rather than health providers.

**Conclusion:**

Focusing on social practices as the unit of analysis offers a more layered understanding of elements in young children’s oral health care that can indicate where the problem may lie. Findings provide an opportunity to consider future research and policy directions in children’s oral health.

**Knowledge Transfer Statement:**

Examining social practices related to young children’s oral health care identifies parents/carers’ knowledge about, for example, toothbrushing, the resources required, and why toothbrushing is important. Analyzing these separate elements can reveal both enablers and barriers to oral health care. This provides researchers, clinicians and policymakers an opportunity to focus on not changing individual behavior but understanding how social context impacts parents/carers’ capacity to make optimum decisions around young children’s oral health.

## Introduction

Theories are “instruments of selective attention” that help to map the social world and explain why things are the way they are, what entities or elements are involved, and what their relationship is to each other (Warde 2014). We adopt a theory-informed social practice approach to explore oral health in young children. Focusing on social practices offers an innovative way to better understand what happens in families (our focus) within which practices exist, change, or become embedded (Spurling et al. 2013).

Social practice theory shifts the focus away from individual behavior to foregrounding social activity by describing important features of the social world we inhabit that are routinely made and reproduced (Nicolini 2013). Conventional approaches to improve oral health continue to focus on biomedical and individualist theories of human behavior change, independent of social context (Watt 2007). This has resulted in parents often feeling blamed for their child’s poor oral health with an ensuing sense of shame (Durey et al. 2017). Approaches that focus on individual behavior appear to have done little to improve oral health in young children at a population level (Albino and Tiwari 2016; Blue et al. 2016). Expecting knowledge alone to change behavior fails to respond to the complexity and challenges of people’s lives, yet such approaches prevail (Bettinghaus 1986).

The social practice approach proposes a shift in emphasis away from the individual and onto the context of people’s lives, activities, and the social relations involved. Practices have been described as routine activities embedded in social relations involving interconnecting elements—mental, physical, and material elements such as motivation, knowing how to carry out the activity, and what materials are needed to perform and reproduce the practice across time and space (Reckwitz 2002; Shove et al. 2012). Engaging in a social practice requires competence, materials or resources, and understanding (meanings) that are linked and integrated in the performance and reenactment of the practice across time and space. Competence and meanings include what to expect from the practice; how to act, speak, and feel; and what things mean and why they matter (Reckwitz 2002; Shove et al. 2012; Nicolini 2013). In the example of toothbrushing, the element of competence reflects knowledge and skills needed to brush teeth, as well as materials and infrastructure involving a toothbrush, toothpaste, and running water for toothbrushing, and meanings include why brushing teeth is important to maintain oral health. All 3 elements are linked and integrated into the performance of brushing teeth (Shove et al. 2012).

By focusing on the practice as the unit of analysis, we move away from an ineffective focus on the individual to explain poor health outcomes (Nicolini 2013; Maller 2015). Shove et al. (2012) suggest that practices emerge, change, or disappear when connections between elements are made, sustained, or broken. Linking the elements is integral to the repeated performance of the practice that becomes embodied as a routine activity that is reenacted automatically without a person being consciously aware of each element (Reckwitz 2002; Shove et al. 2012; Schatzki 2021). Given that enactment of a practice requires the integration of competence, materials, and meanings for it to be reproduced, we are interested not only in which links in current reenactments of oral health practice are likely to facilitate or constrain good outcomes but also in identifying broader factors, such as structural issues, informing the adoption of those links. Researching young children’s oral health from a social practice perspective rethinks the centrality of the social world in promoting or undermining oral health outcomes (Nicolini 2013; Durey et al. 2021).

Social practices, in this context, relate to the routine activities and interactions that may promote or compromise oral health, and they are influenced by structural, social, material, and symbolic factors (Schatzki 1996; Vihalemm et al. 2015). Research on social practices associated with young children’s oral health explores how they are carried out, by whom, when, and where and may address a gap in knowledge regarding the “causes of the causes” of poor oral health (Durey et al. 2021).

While there is no unified theory of social practice, we draw on contemporary approaches to offer a different, more contextualized perspective to understand and explain social practices as they relate to oral health in preschool children (Nicolini 2013; Maller 2015). We suggest that using this developing body of work can provide new insights into how young children’s oral health is understood, organized, and analyzed (Warde 2005).

Although often considered preventable, dental disease remains a global public health problem, impacting across the life course and often leading to pain, infections, lost productivity, delayed growth, and compromised nutrition, cognitive development, concentration, and school participation (Selwitz et al. 2007; Veale et al. 2016; Australian Institute of Health and Welfare [AIHW] 2022). Dental disease (in children, largely dental caries) remains one of the most widespread and costly diseases of early childhood, is often untreated or treated late in its progression due to a lack of oral health education and limited access to dental care, and is exacerbated by the prohibitive cost of care (Mouradian et al. 2000; Selwitz et al. 2007; AIHW 2022). This affects families from socially disadvantaged groups, including children (Peres et al. 2019).

Australia provides a mainly private model of dental care, where about 85% dentists are in private practice and where dental care is focused mainly on individual treatment rather than prevention (Brennan et al. 2008). Expenditure on dental services in Australia is around $10 billion per annum, yet current approaches to education, clinical practice, legislation, and policy have failed to adequately improve oral health outcomes for Australian children at a population level (Do et al. 2010; Cohen et al. 2017; AIHW 2018). Despite being a high-income country, dental disease in young Australian children represents substantial morbidity often associated with preventable hospital admissions that usually require general anesthetic (Alshehri et al. 2021; AIHW 2022).

Much research has been conducted on the negative impact of poor oral health on the quality of life of preschoolers (and parents), being worse in social groups who have been marginalized, including Indigenous, refugee, and disabled children and children living in low-income households (Marques and Vieira-Andrade 2013; AIHW 2022). The Healthy Smiles Healthy Kids longitudinal study in Australia found that socioeconomic disadvantage was the most significant factor associated with early childhood caries (ECC) in preschool children (Manohar et al. 2021). Yet, the food and beverage industries continue to promote processed food high in carbohydrates and sugar content at affordable prices despite the damaging effects on overall health, including dental caries (Cohen et al. 2017). A recent scoping review of Australian preschoolers’ oral health found oral health providers focused more on treating, not preventing, disease where primary carers’ knowledge about the importance of a healthy diet and oral health care practices such as regular dental checkups was inconsistent and limited (Andrew et al. 2021).

Research has identified parents’ frustration with conflicting oral health messages from health professionals (Duijster et al. 2015). It is important for health providers to use a positive approach to provide clear and consistent oral health information, including seeking to understand how social context can inform oral health practice and influence decisions (Bettinghaus 1986; Duijster et al. 2015; Blue et al. 2016). Adopting a multidisciplinary approach where primary health service providers collaborate with oral health professionals to promote oral health may also have the potential to improve oral health outcomes (Lang et al. 2020).

This article’s more holistic approach to improving oral health in young children responds to the question, “What are the practical, social, and material arrangements (social practices) around the oral health of Australian preschool children?” The intent of this work is to move away from a focus on individual behavior change to one that includes the social context in which individual lives are embedded. The aim of this article is to use social practice theory as an analytic framework to investigate preschool children’s oral health in families from a range of socioeconomic backgrounds to better understand the context within which practices exist, change, or become embedded. Rather than a “one-size-fits-all” response, this approach helps us understand which elements of a practice can improve or undermine oral health outcomes.

## Methods

### Design

While there is no specific social practice methodology, we concur with de Souza Bispo (2015), who argues that qualitative researchers develop the most appropriate methodology to meet their research goals—which might involve creating new methods if existing approaches limit their data collection and analysis. We have chosen focused ethnography because it can be applied to a particular issue or shared experience in specific settings with a view to deepening understanding of the context and interrelationships that, in this case, affect young children’s oral health (Cruz 2013). While ethnography broadly describes and interprets a cultural or social group or system, often unfamiliar to the researcher who is involved in prolonged participation in and observation of the daily lives of those being studied (Creswell and Creswell 2018), focused ethnography is “ethnography at home” in research fields familiar to the researcher. It is problem focused and context specific, where participants have particular knowledge of the identified problem. It involves short-term, targeted field visits at various intervals using a range of data collection methods. These include observation, semistructured interviews, and group discussions given the recognized value of such approaches for exploring complex issues where little quantitative research is available (Knoblauch 2005). Focused observations in homes allow researchers to gather more information about practices, materials, and resources than may be reported (intentionally or not) by participants. Confirmability was supported by a team with diverse but relevant expertise from interdisciplinary backgrounds. including clinical dental, dental public health, social science, psychology, and social epidemiology. While all authors are experienced qualitative or mixed-method researchers, 3 authors (A.D., E.H., P.W.) have also conducted research in traditional ethnography.

### Recruitment

The research team established a project advisory group (PAG) by inviting consumers and key stakeholders from policy, oral/dental health, and primary health care to work with and advise the research team from design throughout the project to translation of findings. Our sampling frame focused on metropolitan Perth, Australia. Drawing on the knowledge and expertise of PAG members, approaching parents and carers at playgroups was identified as an appropriate way to access and engage participants in the research. Playgroups were located in the southern suburbs of Perth and engaged parents from different socioeconomic and cultural backgrounds. Management and staff of playgroups were approached by the team and, once they were confident about the nature of the research, provided advice regarding the best way to engage parents. This included placing flyers alongside the attendance register, information on the playgroup’s Facebook page, and the researcher (E.H.) participating in playgroups and starting casual conversations with parents/carers or more proactively bringing morning tea and speaking to the parents/carers as a group, inviting them to participate in the research. A $30 gift voucher was offered to participants. Contact details were established for the parents/carers who expressed interest in participating, and a suitable interview time and place was negotiated to suit participants, including in the participant’s home.

### Data Collection

Data collection involved mainly interviews with opportunities for observation where the interviewer (E.H.) was invited into the home of participants. Questions reflected the *entity* of social practice by identifying *materials, competence, and meanings* participants associate with preschool children’s oral health that underpinned its *performance.* Questions were designed to identify and explore routines and actions associated with parents/family caring for preschool children’s oral health (Shove et al. 2012). They also included brief demographic details, including age (family members and children), family composition, presence of a partner, or family support (see Table 1). We used checking with participants within interviews as a form of member checking to enhance trustworthiness. Following informed consent, interviews were recorded, transcribed, and imported into NVivo12 to assist in the organization and management of data. Interviews were between 45 and 60 min. Observations were made of the home environment and interactions not directly related to the interview questions.

**Table 1.** Participant Demographic Characteristics.

Participant (Pseudonyms)	Participant Age, y	Household Composition	Child Age	Interview Location
Jasmine	40	2 parents, 3 children Full-time mother	11 y, 8 y, 3 y	Participant’s home
Sue	30	2 parents, 2 children Full-time mother	11 y, 3 y	Participant’s home
Liz and Charlie	32 and 31	2 parents, 3 children Parents both work	4 y, 3 y, 11 mo	Participant’s home
Jo	19	2 parents together but living apart. Participant lives with her mother and baby. Participant at school	4 mo	Participant’s home
Karen	32	2 parents, 1 child, and participant’s father Full-time mother	4 y	Participant’s home
Barbara	35	2 parents, 2 children Mother studies and works	8 y, 5 y	Video call to participant’s home
Sharon	35	2 parents, 1 child. Mother works part-time. Grandma helps with childcare.	2 y	Participant’s parents’ home
Wendy	39	2 parents and 2 children Full-time mother	5 y, 3 y	Library
Leila	35	2 parents and 2 children Full-time mother	2.5 y, 4 mo	Participant’s home
Joan	22	2 parents and 2 children Full-time mother	6 y, 2 y	Phone call

### Data Analysis

Interviews with participants were transcribed verbatim, deidentified with pseudonyms, and analyzed by 2 researchers (E.H. and A.D.) to identify key themes emerging from the data. Themes were discussed with the team, reviewed, and then reclassified under the categories of a social practice: *competence, materials, and meanings* that were linked and integrated into its *performance* (Table 2) indicating the practice, not the individual, as the unit of analysis (Nicolini 2013; Maller 2015) (see Fig.). This required classifying themes under the categories of *entity* (competence, materials, meanings) and *performance* (Shove et al. 2012) (see Table 2). Field notes following interviews, including observations from interviews, were also imported into NVivo12 for data management and analyzed to provide some context around the social practice of oral health. Transcripts were also interrogated to identify which themes under specific elements promoted or undermined oral health in the recurrent performance of the practice, noting similarities and differences in participants’ responses. This aspect of the analysis also reflected how participants engaged in the practice, as well as how and when elements of the practice were sustained and enacted routinely, changed, or ceased altogether and factors that informed such changes (Schatzki 1996; Warde 2005; Shove et al. 2012).

**Table 2.** Example of Organising Themes into the Social Practice Theory Framework.

Data Collection	Participant(s)	Social Practice: Competence	Social Practice: Materials	Social Practice: Meanings	Social Practice: Performance
Interview, observation	Pseudonym	Sources of information from: Online groups (Facebook) Mothers’ group Family Health providers	Novelty toothbrushes to mitigate effects of sugar consumption Drink water rather than sugary beverages Healthy diet—home cooking rather than processed foods	Awareness of damaging effects of sugar Electric toothbrushes as motivating factor Water, not fizzy drinks Dental visits—influence of past experiences Cost	Oral health care following eruption of first tooth Oral health care routines facilitated if fun (e.g., use of novelty toothbrushes, phone apps) Patterns of sugar consumption

**Figure.**
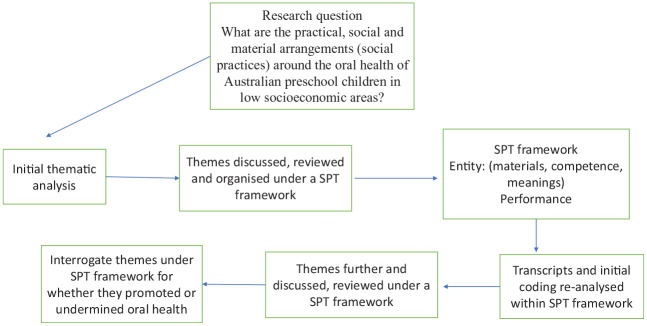
Process of analysis.

### Ethics Approval

Ethics approval for this research was granted by the [The University of Western Australia] Human Research Ethics Committee.

## Findings

Key themes related to young children’s oral health were initially identified and included children’s diet, oral health routines, dental visits, feeling judged, and sources of oral health information. Findings from the reorganization of themes under a social practice framework are presented with social practice as the unit of analysis and where the performance presupposes the integration of the elements that make up the practice (Warde 2005). First, illustrating the *performance* of oral health care such as reducing sugar consumption and then describing the *entity* or elements necessary to do this: competence requires knowing how to reduce sugar consumption and mitigate its effects, materials include drinking water rather than sugar-sweetened beverages and toothbrushing equipment, and meanings and motivations include occasional sugar consumption at children’s parties accepted as a treat, often mitigated by encouraging toothbrushing. These elements are linked together and integrated into the performance of oral health care as a routine practice (Shove et al. 2012) (Table 2). While there will inevitably be some overlap between elements, the analysis focuses on what the *practice* demands, rather than looking through the prism of individual characteristics and behavior (Blue et al. 2016). Habits and routines reflecting social practices are not static but are constantly challenged and transformed in time and space; as children grow and develop, certain elements of the practices become redundant and new ones emerge (Shove et al. 2012).

Nicolini (2013) suggests that a study of a practice starts in the middle of the action, the performance, which is contingent on accomplishing the capacities and competencies required of a carrier of that practice. Participants included 10 mothers (6 had full-time carer responsibilities, 1 was at school, and 3 worked full- or part-time, including 1 participant who was studying) and 1 father. All were either married or partnered, with preschool children of various ages, and were interviewed individually, including 1 participant who invited the researcher into her home to interview her and her friend separately (see Table 1). Interviews conducted in participants’ homes allowed the researcher to observe materials and resources relevant to oral health such as snacks being provided to the children in that setting and the display (by participants) of toothbrushes. The researcher (E.H.) was also able to observe participants’ playgroup settings, noting that playgroups were held in low socioeconomic locations.

Our findings identified oral health care as a social practice within parenting and involved related bundles of social practices such as meals, shopping, family relations, and sugar consumption that were linked and distributed across space and time throughout the day (Reckwitz 2002; Schatzki 2021). Oral health care practices required parents to develop competence and knowledge of materials, as well as often navigating conflicting meanings related to oral health and parenting.

### Performance of Oral Health Care

Findings indicate that oral health involves practices related to diet, shopping, dental visits, social relations, and the performance of routine oral health care for preschool children, such as toothbrushing, often with a fluoridated toothpaste. Performance also reflected participants’ level of oral health literacy, illustrated in awareness of the damaging effects of sugar consumption, ensuring their children drank water rather than sugary beverages, and the importance of routine toothbrushing to maintain oral health. Sugary treats often from grandparents or at children’s parties were indulged as the exception rather than the rule, with participants acknowledging this was not a regular practice at home. The practice of oral health care in some families was established before the child was 1 year old and involved parents cleaning the child’s teeth. Engagement in the practice was contingent on parents knowing the elements of the practice such as when to initiate tooth cleaning, how to do it, which materials to use, and frequency and meanings associated with the practice. Over time, reenacting the performance of these practices became a routine daily activity, thus reproducing a practice to prevent dental caries.

We just did make it routine from very, very, early on, so it was this is just what you do, just you have a bath or a shower every night, you then brush your teeth from 6 months. . . . So it was easy in that regard. (Sue)

We started when they started getting their first tooth . . . that’s when we started brushing. (Joan)

Performing the practice required modification as the child got older and wanted to brush their teeth themselves. Parents’ competence was demonstrated by knowing which elements needed modifying to facilitate this independence, such as allowing the child to improve their skills and practice brushing their own teeth.

She didn’t really like having them brushed, and she started to be quite keen in doing it herself. (Leila)

Brushes his teeth by himself and I’m quite confident. I’ve watched him do it. He’s quite thorough. (Liz and Charlie)

As this performance became a daily routine, the practice was reproduced with this modification. While this was generally supported by parents, some children needed extra help in the transition process.

I do let X brush his own teeth in the morning even though I know he’s not doing a good job. But I think it’s better than nothing and I do it in the night-time, help him do it at night. (Liz and Charlie)

Reenacting the practice as a routine daily activity seemed to reinforce some children’s motivation, occasionally exceeding the twice-daily toothbrushing recommendations.

I don’t know what my secret is and they’re always asking to brush their teeth. . . . He just loves brushing them. (Joan)

This family had started brushing their children’s teeth after the first tooth erupted and it developed into a routine daily activity. Other children were less enthused, with some actively resisting.

She wants to make everything difficult. So, she doesn’t really want a toothbrush, so she’ll put up whatever fight she can. . . . “No, I don’t want that [toothpaste]. I want this one.” (Barbara)

While this family also began toothbrushing when the first tooth erupted, they still brushed their children’s teeth for them. Resistance to toothbrushing occurred in other families but was offset using materials including electric toothbrushes to motivate children to brush their teeth so it became fun.

The electric toothbrush has been a godsend for all the children. So, he’s been using electric toothbrush for about more than 9 months now, and that’s good. (Jasmine)

As the practice becomes routinized, parents and children become “carriers” of the practice (Reckwitz 2002). While some argue that the elements of competence, meanings, and materials are outcomes of being engaged or recruited into the practice (Blue et al. 2016), they are also preconditions for the performance. Mitigating the negative effects of sugar consumption with toothbrushing, for example, requires knowing the materials involved, how to use them, where to buy them, and which are most likely to motivate their child to want to brush their teeth. To be recruited into the practice and know what is involved, oral health literacy is important, including parents knowing where to gather information. Engaging in the practice also requires meanings associated with the importance of oral health. Parents struggling with the competing demands of parenting may consider oral health less of a priority when other issues take precedence.

### Competence and Meanings

Participants were aware that maintaining oral health does not just involve brushing teeth; children often ate home-cooked meals and drank water at home rather than sugar-sweetened beverages, which were mainly consumed as a treat, for example, at parties.

I got brought up with like frozen stuff and you just chuck it in the microwave or the oven . . . I mainly got like frozen pizzas or pies . . . [but] I always make [daughter’s] breakfast, lunch, and dinner . . . I did buy from the shops when she first started just to see how she went and then I started making everything on my own. (Jo)

No parent mentioned the cost of providing a healthy diet as a barrier to maintaining oral health. Oral health was a dispersed practice in the sense that it was located in different sectors of social life, rather than occurring just in one place (Schatzki 2021). Meals and shopping constitute a social practice bundle that is linked to oral health. The damaging effects of sugar causing dental caries are well documented (Peres et al. 2019), and generally, participants avoided sugar-sweetened beverages.

I think that was the biggest thing, is not having juice in the house. I think if we do for whatever reason, it’s just—they just go crazy for it. So we try not to even have it here. (Liz and Charlie)

Given the often-negative effects of behavior associated with the consumption of products with added sugar, water was the preferred option over sugary drinks for babies who were being weaned off the breast:

I haven’t given her anything besides water. I’m scared to, ’cause I don’t want her to get addicted to it and then she’ll have a tantrum over it. (Jo)

However, most parents were less inclined for their children to avoid sugar altogether, accepting it was pleasurable and seen as a treat, rather than a routine practice.

I also didn’t really wanna deprive them of sugar because of the whole rebound thing that I’ve heard about so they have it in small doses. (Wendy)

Several participants commented that their own parents were supportive of their grandchildren’s routines around toothbrushing.

With my mum, it would be if I said “mum just brush his teeth in the morning,” I probably would give her my toothpaste that we have. And I imagine with my mother-in-law, it would be the same thing, they would ask me, “What do you do?” I’ll say, “Do this,” if he does it, as long as he gives it to go. (Sharon)

One mother commented that while her children regularly consumed sugary snacks, consumption of sugary drinks was rare.

So they do eat, I reckon, too many treats, but it’s generally after school . . . they do have a lolly or a piece of chocolate after school or in the evening, most nights. . . . Soft drink and juice, they very rarely get. It’s always water, sometimes milk. (Barbara)

While most participants seemed to accept, rather than forbid, occasional consumption of sugary products, they were also well informed about how to mitigate its damaging effects:

When she got to the age where she was starting to have lollies and things like that, it was more like, “Okay, we have to brush her teeth,” to the point where it’s like, “Okay, you can’t have any lollies tomorrow at all if you don’t brush your teeth tonight.” (Leila)

However, not all brushed with fluoride toothpaste because of their children’s “sensitivity” (Jasmine) so “natural” nonfluoridated toothpaste was preferred instead, whereas others chose “anything with fluoride because of research” (Sue). Most parents weighed up the importance of allowing their children to enjoy sugary products on special occasions rather than banning it altogether. They offset its damaging effects with information that was linked to practice about how to care for their children’s teeth.

This raised questions about where participants received their information about the importance of oral health. Findings suggest often ambivalent attitudes to traditional sources involving dentists, general medical practitioners, and maternal and child health (MACH) nurses. Several mothers indicated that relationships with health professionals were important yet were sometimes found wanting. Responses to dentists ranged from being “really good” and the children “loved going to see her” (Liz and Charlie), to respecting and trusting dentists’ knowledge, to seeing them as being “just in it for the money” (Jasmine), suggesting a distrust of some health professionals’ motives around care. Despite some participants’ traumatic dental experiences in childhood, they still sought professional dental help if their child needed care.

While regular dental visits were recommended, they were often costly.

Also here, it seems so expensive. That’s another aspect which I haven’t even mentioned. It’s like $200 just to have a tooth cleaned. (Wendy)

To offset the cost of dental visits, this mother ensured her children ate a healthy diet and performed regular oral health care.

I mean we brush their teeth every day and we’re always looking at their teeth and healthy gums, and plus, they don’t have that much rubbish. . . . My sense is I’ll take her when I think there’s an issue. (Wendy)

While waiting for a problem to arise might be too late in terms of prevention, information about oral health care was often sought elsewhere.

Most responses related to information from MACH nurses, with 1 participant stating the nurse was “very helpful” (Joan) and a “bloody useful resource and a good service” (Sharon). Others were more hesitant, particularly around the amount of information received:

They’ve given you so much information to make sure you’re doing this, have these 20 pamphlets that if you were relying on that solely, it would just be overwhelming. (Sue)

One mother expressed her frustration at the attitude and practice of some MACH nurses and considered the information they gave unhelpful:

I’ve not found them friendly. . . . They give outdated and incorrect advice. It doesn’t match what the current recommendations are. And they’re very judgmental. (Jasmine)

Most participants’ primary sources of information instead were mothers’ groups and online support, not just for oral health but also for parenting issues in which the social practice of oral health care was embedded. Learning what was needed for their preschoolers to maintain their oral health was a topic of significant interest.

I’d say a lot of us get most of our info from online these days or from mums’ groups on Facebook. A lot of us are part of at least one Facebook mum’s group. So, things like [information about] the electric toothbrushes and stuff, they would come from something like that. (Jasmine)

Specifically, health wise, I think first thing, we go is online, even before we go to the doctor. So we have a bit of an idea as to what could be the issue. (Karen)

Factors that appealed about mothers’ groups, including those online, were the nonjudgmental and welcoming environment they offered. Mothers felt safe to explore issues of concern related to parenting generally where opportunities for friendships could develop.

I feel very lucky that I joined a mothers’ group and made good friendships and that you could confide in these people and ask for advice. (Barbara)

While such social relations were important, some participants did express the need for caution about uncritically accepting information on social media and noted the importance of considering evidence-based advice.

Facebook groups have almost replaced the child health nurse, which is really concerning because misinformation and fear and all sorts of rubbish can also be spread if it’s not coming from an evidence-based perspective. (Sue)

Most groups I’m in are like—they have professionals in their groups. (Jo)

While mothers learned about oral health care from this environment, misinformation was a risk.

We’re a bit worried about the fluoride as a lot of other mums say that you shouldn’t get it. . . . A lot of other people say that using it, it would ruin your taste more. (Joan)

Some parents were more comfortable brushing their child’s teeth with fluoride as they got older.

Yeah, and I’m all for it . . . the thing I’m worried about at the moment is that he doesn’t spit . . . so if we would have put water in his mouth, he would 100% swallow it. So I guess I’m holding off on anything fluoride-y until he’s at the age where he can understand that he needs to spit. (Sharon)

The ones we use have fluoride . . . I think that it’s probably not that big of an issue given the amount of toothpaste we use. I was aware of the fact that you only should use a small amount. I don’t know how I came across that. Probably . . . when I was Googling how to get your kid to brush their teeth. (Leila)

Recruitment into the practice of oral health required parents accessing information to become competent in learning what elements were needed to perform the practice and embed it into a daily routine so it was reproduced. Parents also gathered information from their own lived experience of oral health routines growing up, with some participants currently also seeking information from family members. From some, oral health routines were embedded intergenerationally as a routine practice.

We have dentists in the family so the oral care and looking after teeth is an ingrained part of our family culture. (Sue)

Materials and motivations as well as competence are also required for recruitment into the practice to initiate the performance of oral health care.

### Materials and Motivations

Recruitment into some oral health practices, initiated before the baby was 1 year old, required specific competence and materials for when the first tooth erupted. Most rejected traditional advice from MACH nurses about introducing solids and opted for baby-led weaning where most information was sourced from social media about how and when to introduce solid foods (materials) into the baby’s diet and when to clean the baby’s teeth.

We started when he first got teeth . . . probably about five-ish months, and we bought a silicone finger brush that we attempted to use a couple of times, so that was our first introduction . . . he would maybe occasionally pop it in his mouth, but actually early on we would use a banana-shaped toothbrush, like a teether, and that had bristles on the end so it looked like a toothbrush. It was silicon and he loved that. He would chew on that. (Sharon)

As the child grew older, materials related to oral health care were modified to include toothbrushes. If a child needed coaxing to brush their teeth, parents were required to expand their knowledge, which sometimes included purchasing novelty toothbrushes. These were popular, as they were fun, particularly with young children who needed motivating with toothbrushes representing a

monkey or a hippo . . . and the most recent one that we’ve bought that we’ve been using for about probably 2 or 3 months is a Jack and Jill one. (Sharon)

Electric toothbrushes were particularly popular in some families and facilitated the routinized practice.

The electric toothbrush has been a godsend for all the children . . . all of them hated the handheld. As soon as you gave them the electric, they were happy. (Jasmine)

Yeah, but she does want an electric toothbrush so she asking for one for Christmas and she will get one. (Barbara)

Other materials that delighted children and motivated them to brush their teeth were phone apps using their favorite characters.

It started off with “Elmo brush your teeth” . . . which is quite catchy, and they’re 2-minute songs, so Elmo’s 2 minutes. Now we’re doing Christmas carols ’cause he loves Christmas. (Sharon)

However, while most participants did not mention cost of materials as a problem to maintaining oral health, for those whose children were particularly sensitive due to comorbidities, it was a barrier. While oral health and shopping were linked bundles of practices, the choice of materials or products to buy depended on other elements such as the meanings parents associated with, for example, toothpaste. While this could include whether a brand contained fluoride, the decision was also contingent on parents’ knowledge of products and meanings associated with the use of fluoride. Nonfluoride toothpastes were generally used when oral health care was initially introduced, although they were more expensive.

I think initially we started off with the organic like all natural products and I don’t really know that I felt like they were doing a good enough job. . . . And we ended up just going for mainstream Colgate or whatever and it was fine. (Wendy)

Convenience and cost were also motivating factors to purchase the product.

Well, I mean the toothpaste is only two bucks, so I’m just kind of, “That works.” I mean I don’t really think I’ve ever bought them on special, but they’re just freely available on supermarkets. (Wendy)

However, for 1 mother whose child was sensitive to toothpaste, cost was a significant issue.

So it’s also just very expensive trying one after another. This is probably the sixth one we’ve tried. So I have spent so much money on toothpaste now. (Jasmine)

Despite this, all participants were recruited into and reproduced the social practice of oral health care as part of their preschool children’s daily routines. Differences between participants manifested in the elements of the practice, for example, information around the use of fluoride or cost of products. These activities linked “bundles” of social practices around meals and snacks and shopping to that of oral health care.

## Discussion

Viewing oral health through a social practice lens allowed us to move away from focusing on individual behavior change to understanding the social context of often inexperienced parents in which young children’s oral health is embedded. This includes identifying the social relations involved and the elements that are linked and integrated into the performance of the practice that is reproduced or changed as the child develops. This approach also highlights how a more layered analysis of factors involved in young children’s oral health avoids a tendency to judge parents for the state of their child’s oral health and to focus more on factors informing how they make decisions and develop practices. It allows links between the elements to emerge that can reflect broader, structural factors, such as negative responses from health providers or cost of treatment that may erode trust and confidence and/or compromise oral health outcomes. It also notes the challenges of developing competencies across many practices involved in parenthood.

Findings demonstrate the social practice of oral health care in parenting young children and the elements required to engage in the practice, accomplish its performance, and reproduce it as a routine activity (Reckwitz 2002; Nicolini 2013). Some participants engaged in the practice often following the eruption of their baby’s first tooth. Practices changed and were reproduced as their children grew and required different materials and competencies to reflect this and maintain their oral health. The social practice of oral health care was performed as a routine informed by understanding how to prevent oral disease. This included avoiding sugar-sweetened beverages or how to mitigate the damaging effects of sugar consumption.

Materials and meanings were elements that also varied within practices and highlighted how practices interconnected; the purchase and use of fluoride toothpaste linked the practices of shopping and oral health care where purchasing electric or novelty toothbrushes (materials) was used as a motivating factor to encourage children to routinely perform oral health care (competence) or maintain oral health following the consumption of sugar (meaning).

Findings also noted that competence to perform the practice was gained from various sources with most participants preferring social media, mothers’ groups, or family members, where they felt welcomed, safe, and respected over seeking advice and support from health professionals, some of whom they distrusted and considered out of touch and judgmental. While other evidence indicates perceptions of an underfunded public health system in Australia rather than the behavior of individual health providers that contributed to a lack of trust in health services (Ward et al. 2015), our findings suggest some participants felt disrespected and judged by individual health providers, constituting a barrier to accessing services and reflecting a structural problem.

Despite recruiting from playgroups in low socioeconomic status areas, cost was not identified as a barrier to providing a healthy diet, although cost of dental products and the high cost of dental services were noted by some participants (Australian Bureau of Statistics 2011). Focusing on the social practice as the unit of analysis helped us identify links in the practice of caring for young children’s oral health that contribute to compromising or promoting oral health. These include noting structural elements that can influence the capacity of parents/carers to make choices around their child’s oral health such as attitudes of health professionals (Watt 2007; Cohen et al. 2017), anxiety over general parenting competency, conflicting information sources, and cost of dental care.

Findings indicate that preschool children’s oral health care involves practices such as consumption of meals and brushing teeth that are dispersed at different times and may involve different social relations such as grandparents (Warde 2005; Schatzki 2021). Both require links to shopping for specific products like toothbrushes that are bundled together and linked to the practice of oral health care. Many participants were aware of the link between sugar and poor oral health and sought to mitigate its negative effects by providing water rather than sugar-sweetened beverages.

An interesting finding was the role of social media and connection to mothers’ groups in seeking information, where participants felt accepted and welcomed, rather than judged, as reflected in some visits to health professionals. This raises questions from a health perspective about the quality of communication and delivery information by health providers to patients/clients to ensure it is respectful and appropriate for the level of oral health literacy and fosters rather than undermines trust. These findings suggest that refocusing the lens onto structural factors, such as those over which individuals have little control, can help guide a policy and practice approach that focuses more on ways to engage rather than alienate patients/clients in the oral health encounter (Watt 2007).

Assumptions that delivering evidence-based knowledge about how to improve their children’s oral health will automatically translate into better practices have proved relatively ineffective (Baum and Fisher 2014). Nonetheless, failure to follow such advice still risks parents being blamed for noncompliance despite evidence that at least some of the problem is the judgmental attitudes of health providers (Durey et al. 2017). Current neoliberal discourse often places the burden of risk on the individual, who is held responsible for making optimum health choices regardless of their social context and constraints (Henderson et al. 2009). Perpetuating such an approach keeps the lens focused on blame and individual choice (Durey et al. 2016), rather than critically interrogating broader structural changes or socioeconomic factors that can undermine parents making optimum health decisions for their children (Watt 2007; Henderson et al. 2009). Current approaches to education, legislation, and policy have failed to adequately address dental disease in Australian children (Do et al. 2010). Doing more of the same is not an option if oral health outcomes are to improve.

Focusing on social practice as the unit of analysis allowed us not only to identify the elements involved but also to consider what social or structural factors informed decision-making that underpinned each element—for example, what sources of oral health information were accessed (competence), whether parents could afford the cost of dental visits (materials), and contested meanings on whether and when to use fluoride. Adopting a social practice lens offers an innovative way to research and understand oral health from a different perspective, where policymakers and health providers can learn about the context in which oral health care is practiced. Social practice as the unit of analysis in research allows us to examine context and factors that influence, for example, parents’ capacity to gain the competence and materials required to perform oral health care and be carriers of that practice.

Blue et al. (2016) state that elements are not evenly distributed across society, and becoming a carrier of a practice depends on what the practice demands in terms of competence, materials, and meanings. Addressing preschool children’s oral health from a social practice perspective offers policymakers and practitioners the opportunity to consider how best to support families’ capacity to engage with and integrate the elements of the practice (competence, materials, and meanings) into its performance. Not having that capacity because, for example, of feeling judged by health providers, peers, or family members, or an inability to afford visits to the dentist can compromise good oral health outcomes. These structural factors are beyond individual control to change yet highlight the importance of considering notions of equity and how structural factors can affect either improving or compromising young children’s oral health (Watt 2007; Cohen et al. 2017).

### Limitations

This research offers a theoretical and methodological approach focusing on the social practice of oral health care as the unit of analysis rather than more conventional approaches involving biomedical and individualist theories of human behavior change. Most families participating in this study appeared to highly value the oral health of their children, suggesting that the research did not capture those families who may have other current or more pressing priorities than the oral health of their children. The research was focused on mothers rather than other family such as fathers and grandparents.

## Conclusion

Locating the oral health of preschool children within a social practice framework shines the lens on the practice as the unit of analysis, seeking to understand young children’s oral health from a contextual and more holistic perspective. This approach to analysis moves away from focusing on individual behavior to a layered understanding of elements in oral health care that can indicate where the problem may lie. Findings can offer researchers, policymakers, and practitioners a deeper understanding of families’ decision-making around oral health to better inform improvements to young children’s oral health outcomes.

## Author Contributions

A. Durey, contributed to conception, design, data acquisition, analysis, and interpretation, drafted and critically revised the manuscript; P. Ward, S.R. Baker, H. Calache, L. Slack-Smith, contributed to conception, design, data analysis and interpretation, critically revised the manuscript; E. Haynes, contributed to design, data acquisition, analysis, and interpretation, critically revised the manuscript. All authors have their final approval and agree to be accountable for all aspects of work.
